# Supplementary material for: Bacterial Communities in the Feces of Laboratory Reared Gampsocleis gratiosa (Orthoptera: Tettigoniidae) across Different Developmental Stages and Sexes
Source: Insects. 2022 Apr 7;13(4):361. doi: 10.3390/insects13040361 (PMC9024567; doi:10.3390/insects13040361)
Supplement: Supplementary file 1 [file insects-13-00361-s001.zip › Table S3. Nonparametric Kruskal-Wallis tests among groups.pdf]

Table S3 Nonparametric Kruskal-Wallis tests

	ACE	Chao1	Shannon	Gini-Simpson
Among different developmental stages of male	chi-squared = 4.5921, df = 3, p-value = 0.2042	chi-squared = 5.6028, df = 3, p-value = 0.1326	chi-squared = 0.26827, df = 3, p-value = 0.9659	chi-squared = 0.27392, df = 3, p-value = 0.9649
Among different developmental stages of female	chi-squared = 3.8443, df = 3, p-value = 0.2788	chi-squared = 4.7754, df = 3, p-value = 0.189	chi-squared = 3.2828, df = 3, p-value = 0.35	chi-squared = 4.3985, df = 3, p-value = 0.2215
Among different developmental stages and sexes	chi-squared = 8.6308, df = 7, p-value = 0.2803	chi-squared = 10.51, df = 7, p-value = 0.1615	chi-squared = 6.8006, df = 7, p-value = 0.4499	chi-squared = 7.1247, df = 7, p-value = 0.416
