# Supplementary material for: Bacterial Communities in the Feces of Laboratory Reared Gampsocleis gratiosa (Orthoptera: Tettigoniidae) across Different Developmental Stages and Sexes
Source: Insects. 2022 Apr 7;13(4):361. doi: 10.3390/insects13040361 (PMC9024567; doi:10.3390/insects13040361)
Supplement: Supplementary file 1 [file insects-13-00361-s001.zip › Table S4. Nonparametric paired t-test between groups.pdf]

Table S4 Nonparametric paired t-test between groups

	ACE	Chao1	Shannon	Simpson
MN12 vs FN12	t = -0.1821,<br>df = 5,<br>p-value = 0.8627	t = -0.13469,<br>df = 5,<br>p-value = 0.8981	**t = -3.0588,<br>df = 5,<br>p-value = 0.02814**	t = -2.3721,<br>df = 5,<br>p-value = 0.06378
MN35 vs FN35	t = 1.05,<br>df = 8,<br>p-value = 0.3244	t = 1.482,<br>df = 8,<br>p-value = 0.1766	t = 0.13188,<br>df = 8,<br>p-value = 0.8983	t = 0.37082,<br>df = 8,<br>p-value = 0.7204
MN67 vs FN67	t = -2.1948,<br>df = 5,<br>p-value = 0.07962	t = -2.2254,<br>df = 5,<br>p-value = 0.07661	t = -0.93133,<br>df = 5,<br>p-value = 0.3944	t = -0.19365,<br>df = 5,<br>p-value = 0.8541
MA vs FA	t = -1.0342,<br>df = 11,<br>p-value = 0.3232	t = -1.1995,<br>df = 11,<br>p-value = 0.2555	t = -1.0452,<br>df = 11,<br>p-value = 0.3184	t = -0.56459,<br>df = 11,<br>p-value = 0.5837
Male vs Female	t = -1.1689,<br>df = 32,<br>p-value = 0.2511	t = -1.1965,<br>df = 32,<br>p-value = 0.2403	**t = -23.79,<br>df = 32,<br>p-value < 2.2e-16**	t = -0.73611,<br>df = 32,<br>p-value = 0.467

Note. Nonparametric paired *t*-test only revealed significant differences in the Shannon diversity indices between Male vs. Female ($P < 2.2e-16$) and MN12 vs. FN12 ($P < 0.05$).
